# M. Dance on a Species of Intermitting Tetanus

**Published:** 1831

**Authors:** 


### M. Dance on a species of Intermitting Tetanus.

We doubt much the propriety of the term, tetanus, applied by M. Dance. However, we shall briefly state the particulars of four cases which the author has detailed in the Archives Générales, for June last, by which the reader will be able to judge for himself.

*Case* 1. A female, 25 years of age, was admitted into the Hôtel Dieu on the 5th October, 1824. She had enjoyed good health, and borne a child without any accident. A short time after her accouchement she was attacked with a kind of fit, characterized by numbness of several parts of the body, and followed by stiffness in the limbs, resembling painful cramps, which caused the patient to cry out; while the skin was hot, and covered with perspiration, the pulse hard and frequent. These attacks took place irregularly, and lasted three or four hours—latterly much longer. The patient, therefore, entered the Hôtel Dieu, when it was observed that the fore-arm was semi-flexed—the hands clenched—the lower limbs extended rigidly, requiring much force to bend them. The febrile symptoms induced the medical officers to have recourse to venesection, which rather augmented than diminished the symptoms, as indeed it had done before entering the hospital. On the 6th and 7th these attacks came on with great violence, and various means were used, including the hot bath, fomentations, frictions, &c. On the 8th an unusually severe paroxysm occurred, lasting all day, and not at all relieved by the bath, &c. In the evening, a slight catamenial discharge took place, and a dozen of leeches were applied to the thighs. After this, no attack occurred till the 15th, when a mild one came on, and lasted for three days, *accompanied by symptoms of hysteria.* She then got quite well, and soon left the hospital.

The author concludes that the above could not be a case of anomalous hysteria—because febrile symptoms never occur in this complaint. "Dans l'hysterie, quelques variés que soient ses symptômes, il y a *toujours* absence de fièvre." We say, no. Fever is not exempted from being pressed occasionally into the interminable catalogue of hysterical affections.

*Case* 2. A young steel-polisher had been subject for two years to attacks somewhat resembling those noticed in the female patient. He was admitted into the Hôtel Dieu on the 21st of March, 1826, and at 10 o'clock the same evening he was assailed with stiffness of his limbs, which continued to increase till the fore-arms were rigidly flexed, the fingers clenched and the lower extremities immoveably extended. All these parts were very painful—his face was flushed—he was covered with perspiration — thirst urgent — pulse full, hard, and frequent. He continued in this state throughout the night. 22d. The flushing of face and the agitation increased. He desired to be bled, and his wish was complied with. No relief followed. In the evening, the same affection of the extremities returned, together with spastic rigidity of several muscles on the trunk and about the head. He was put into a warm-bath, which increased rather than diminished the symptoms. From this time, however, the intensity of the phenomena gradually subsided, and the man left the hospital on the 18th of April. He had no relapse.

*Case* 3. A goat-keeper, aged 46 years, had just emerged from an imprisonment of half a year's duration, when he was abruptly seized with stiffness and numbness of his limbs. In this state he continued for about a fortnight, with occasional exacerbations and mitigations of the complaint. On the 2d of Feb. 1826, he was attacked more violently than usual, with spastic contraction of the arms, and, at the same time, numbness of the lower extremities, lasting some hours, accompanied by re-action and perspiration. A general sense of soreness followed. The next day was passed without complaint. On the 4th he repaired to the Hôtel Dieu, where he also spent a quiet day. In the middle of the following night, however, he was visited by the same train of symptoms, and when visited at seven o'clock next morning, he was found immoveable as a statue, the fore-arms bent rigidly, the fingers clenched, the lower extremities extended and rigid, the muscles of the thigh and calf of the leg hard and tense as though they were violently cramped. The muscles of the chest and neck were unaffected, but the jaws were firmly closed. The pain was severe in the contracted muscles, the pulse was quick, and perspiration covered the body. The intellects were undisturbed. By the evening the paroxysm had subsided in a deluge of perspiration. No treatment was employed, and no attack returned, though the man was kept in the hospital for some months as an assistant.

We cannot view the two last cases as other than anomalous intermittents having nothing to do with tetanus. The 4th case does not differ materially from those we have narrated. The paroxysms were intermittent, and the cure was soon effected by quietude and regimen, in the hospital, without any medicine.

